# COVID-19: association of the National Early Warning Score with triage categories, severity and outcomes

**DOI:** 10.1590/0034-7167-2024-0069

**Published:** 2025-08-08

**Authors:** Luiz Humberto Vieri Piacezzi, Jaine Novaes da Silva, Rui Carlos Negrão Baptista, Karina Aparecida Lopes da Costa, Maria Carolina Barbosa Teixeira Lopes, Ruth Ester Assayag Batista, Cássia Regina Vancini Campanharo

**Affiliations:** I Universidade Federal de São Paulo. São Paulo, São Paulo, Brazil; II Escola Superior de Enfermagem de Coimbra. Coimbra, Portugal

**Keywords:** Triage, Early Warning Score, COVID-19, Emergency Medical Services, Nursing Care., Triaje, Puntuación de Alerta Temprana, COVID-19, Servicios Médicos de Urgencia, Atención de Enfermería.

## Abstract

**Objectives:**

to identify the association of the National Early Warning Score (NEWS2) and NEWS Age with risk categories, severity markers and outcomes in the emergency department.

**Methods:**

retrospective cohort study, conducted in a high-complexity hospital, with 356 hospitalized patients (mean age 59.4; ±14.4 years) with COVID-19, from April to August 2020. To verify the association between risk categories and alert scores, the chi-square, Kruskal-Wallis and likelihood ratio tests (p<0.05) were used.

**Results:**

patients stratified in the red category had higher NEWS2 and NEWS Age (<0.0001) than the others. Clinical risk categorized by scores was associated with clinical deterioration (p<0.0001), orotracheal intubation (p<0.0001) and death (NEWS - p=0.0098/NEWS Age - p<0.0001).

**Conclusions:**

scores ≥ 7 were associated with red/orange risk stratification, clinical deterioration and occurrence of death.

## RESUMEN

**Objetivos:**

identificar la asociación del *National Early Warning Score* (NEWS2) y NEWS Age con categorías de riesgo, marcadores de gravedad y resultados en el servicio de urgencias.

**Métodos:**

cohorte retrospectiva, realizada en un hospital de alta complejidad, con 356 pacientes ingresados (edad media 59,4; ±14,4 años) con COVID-19, de abril a agosto de 2020. Para verificar la asociación entre categorías de riesgo y puntajes de alerta se utilizaron las pruebas de chi-cuadrado, Kruskal-Wallis y razón de verosimilitud (p<0,05).

**Resultados:**

los pacientes clasificados en la categoría roja tenían NEWS2 y NEWS Age (<0,0001) más altos que los demás. El riesgo clínico categorizado por los puntajes se asoció con deterioro clínico (p<0,0001), intubación orotraqueal (p<0,0001) y muerte (NEWS - p=0,0098/NEWS Edad - p<0,0001).

**Conclusiones:**

puntuaciones ≥ 7 se asociaron con clasificación de riesgo rojo/naranja, deterioro clínico y ocurrencia de muerte.

## INTRODUCTION

The Coronavirus Disease (COVID-19) pandemic has brought great challenges to healthcare professionals, especially emergency departments (ED), which are the gateway to the Brazilian Health System (In Portuguese, *Sistema Único de Saúde* - SUS)^(1)^. These units faced overcrowding and rapid clinical deterioration of patients^(2)^. The negative effects of overcrowding on patient quality and safety are well established and documented. A retrospective cohort study of 995,379 admissions to the ED in 187 hospitals showed that patients treated during periods of overcrowding were five times more likely to die^(3)^.

COVID-19 infection can range from a simple cold to severe pneumonia, leading to severe acute respiratory syndrome, circulatory shock and death^(4,5)^. In view of this situation, it is essential that the health team, especially nursing staff, develop skills to perform assessments that identify clinical deterioration in these patients early so that therapeutic measures can be adopted early, preventing unfavorable outcomes. The use of early warning scores for clinical deterioration has shown good performance in hospital settings, reducing mortality and improving the survival of these individuals^(6,7)^.

The National Early Warning Score (NEWS2) and NEWS Age can help identify clinical deterioration in adult patients. Studies show that using NEWS2 when admitting patients with COVID-19 to the ED can identify potential clinical complications associated with the disease early^(7,8)^. However, especially on the national scene, literature is scarce.

Given the above, concern about quality of care in the ED has intensified, and it has become pertinent to investigate the relationship between early warning scores, risk categories and outcomes of patients with COVID-19, given that these tools minimize unfavorable outcomes and promote health safety.

## OBJECTIVES

To identify the relationship between NEWS2 and NEWS Age with risk categories, severity markers and outcomes in the ED.

## METHODS

### Ethical aspects

The study was guided by Resolution 466/2012 and approved by the *Universidade Federal de São Paulo* Research Ethics Committee. The Informed Consent Form was obtained online. Identification data were anonymized and replaced by tags.

### Study design, period and location

This is a retrospective cohort study conducted at a hospital called *Hospital São Paulo* (HSP), a large, highly complex hospital that primarily serves SUS patients. HSP is responsible for covering an area with over five million inhabitants, in addition to serving patients from other states, and was a reference for treating COVID-19 cases^(9)^. The methodological steps were in accordance with STrengthening the Reporting of OBservational studies in Epidemiology (STROBE) guidelines^(10)^.

### Population: inclusion and exclusion criteria

The population consisted of 356 patients, over 18 years old, treated at the HSP ED, diagnosed with COVID-19 and who remained hospitalized from April to August 2020. Pregnant women, patients in palliative care and patients with incomplete data for calculating the scores were excluded.

### Study protocol

Data were obtained through analysis of electronic medical records; for this purpose, a collection instrument was developed using the Research Electronic Data Capture (RECDap^®^) application, consisting of sociodemographic variables, comorbidities, signs and symptoms, risk category, vital signs for calculating NEWS2 and NEWS Age on admission, occurrence of clinical deterioration (CD) (acute respiratory failure (ARF), shock and cardiopulmonary arrest (CPA), severity markers (need for prone position, mechanical ventilation and length of hospital stay) and outcomes), discharge, death, or transfer^(11)^.

The Reception with Assessment and Risk Stratification at HSP uses five categories, which indicated the recommended waiting time for medical care: red (immediate care); orange (ten minutes); yellow (60 minutes); green (120 minutes); and blue (240 minutes)^(12)^. NEWS2 consists of heart and respiratory rate, oxygen saturation, need for oxygen support, temperature, systolic blood pressure, and level of consciousness^(13)^. In the case of NEWS Age, the calculation is made by adding 3 points for patients over 64 years of age^(14)^. Individuals are categorized into low (0-4 points), low-medium (3 on any parameter), intermediate (5-6) and high (≥ 7) clinical risk^(13)^.

### Analysis of results, and statistics

For continuous variables, mean, standard deviation, median, minimum and maximum were calculated. Regarding categorical variables, frequency and percentage were calculated. To compare categorical variables with clinical risk of NEWS2 and NEWS2, the chi-square test was used and, when necessary, the likelihood ratio test. To compare continuous variables with clinical risk of NEWS2 and NEWS2, the Kruskal-Wallis test was used. A significance level of 5% (p-value < 0.05) was used. The analyses were performed using the Statistical Package for the Social Sciences^®^ software.

## RESULTS

The mean age of the population (n=356) was 59.4 years (±14.47), with the majority being male (n=226-63.7%), white (n=181-51.4%), with incomplete elementary education (n=105-37.4%) and with comorbidities (n=304-85.4%). The most frequent comorbidity was hypertension (n=211-59.3%), followed by Diabetes Mellitus (n=137-38.5%), heart disease (n=76-21.3%) and solid organ transplant (n=70-19.7%).

The most frequent symptoms were dyspnea (n=246-69.1%), cough (n=244-68.5%) and fever (n=223-62.6%), with the mean number of days from symptom onset to seeking care being 7.48 (±4.05) days. As for risk category, 135 (37.9%) patients were stratified in the orange category, 103 (28.9%) in the yellow category, 71 (19.9%) in the red category, 46 (12.9%) in the green category, and one (0.3%) in the blue category. When clinical risk upon admission to the ED was calculated, the majority presented “high risk” according to NEWS2 (n=53.4%) and NEWS Age (n=67.1%).

Most patients (n=205-57.6%) presented CD, and in 61.4% (n=126), this occurred within the first 24 hours after admission to the ED. The most frequent first CD was ARF (n=195-54.8%), and during hospitalization, it was shock (n=116-32.6%). Most patients did not require prone positioning (n=267-75.0%) or mechanical ventilation (n=228-64.0%). The mean length of hospital stay was 13.46 (±13.98) days. Hospital discharge (n=236-66.3%) was the most frequent outcome.

Patients stratified in the blue/green and yellow categories had a higher proportion of low and low-medium risk according to NEWS2 than those stratified in the orange and red categories, which had a higher proportion of high risk (p<0.0001) (Table 1).

**Table 1:** Association of clinical risk, according to the National Early Warning Score, with risk category, clinical deterioration, severity markers and outcomes, São Paulo, São Paulo, Brazil, 2020

	Clinical risk according to NEWS2 n(%)				Total	*p* value
	Low n=45	Low-medium n=23	Medium n=98	High n=190	n=356	
Risk category						
Blue/green	8(17.8)	9(19.1)	18(38.3)	12(25.5)	47(13.2)	<0.0001^**^
Yellow	27(26.2)	5(4.9)	40(38.8)	31(30.1)	103(28.9)	
Orange	8(5.9)	9(6.7)	28(20.7)	90(66.7)	135(7.9)	
Red	2(2.8)	-	12(16.9)	57(80.3)	71(19.9)	
CD						
Yes < 24 hours	6(4.8)	6 (4.8)	28(22.2)	86(68.3)	126(35.4)	0.0001^**^
Yes > 24 hours	7(8.9)	6(7.6)	23(29.1)	43(54.4)	79(22.2)	
No	32(21.2)	11(7.3)	47(31.1)	61(40.4)	151(42.4)	
1st CD - ARF						
Yes	11(5.6)	12(6.2)	51(26.2)	121(62.1)	195(54.8)	<0.0001^**^
No	34(21.1)	11(6.8)	47(29.2)	69(42.9)	161(45.2)	
Other CDs						
Yes	10(7.2)	7(5.1)	23(16.7)	98(71.0)	138(38.8)	<0.0001^**^
No	35(16.1)	16(7.3)	75(34.4)	92(42.2)	218(61.2)	
Other CD (ARF)						
Yes	8(8.4)	6(6.3)	17(17.9)	64(67.4)	95(26.7)	0.012^**^
No	37(14.2)	17(6.5)	81(31.0)	126(48.3)	261(73.3)	
Other CD (shock)						
Yes	8(6.9)	6(5.2)	20(17.2)	82(70.7)	116(32.6)	0.0001^*^
No	37(15.4)	17(7.1)	78(32.5)	108(45.0)	24(67.4)	
Other CD (CPA)						
Yes	7 (7.3)	4 (4.2)	20 (20.8)	65 (67.7)	96(27)	0.0105^**^
No	38 (14.6)	19 (7.3)	78 (30.0)	125 (48.1)	260(73)	
Length of hospitalization						
Mean (SD)	9.6 (8.5)	12.17 (9.9)	11.5 (13.1)	15.5 (15.5)	13.4 (13.9)	0.0162^*^
Median (min-max)	7(1-40)	9(1-37)	8(0-80)	11(1-98)	8(0-98)	
TI/MV						
Yes	10 (7.8)	6 (4.7)	21 (16.4)	91 (71.1)	128(36)	<0.0001^**^
No	35 (15.4)	17 (7.5)	77 (33.8)	99 (43.4)	228(64)	
Prone position						
Yes	5 (5.6)	5 (5.6)	16 (18.0)	63 (70.8)	89(25)	0.0015^**^
No	40 (15.0)	18 (6.7)	82 (30.7)	127 (47.6)	267(75)	
Final outcome						
Discharge	36 (15.3)	18 (7.6)	70 (29.7)	112 (47.5)	236(66.3)	0.0098^**^
Death	8 (8.0)	5 (5.0)	19 (19.0)	68 (68.0)	100(28.1)	
Transfer	1 (5.0)	-	9 (45.0)	10 (50.0)	20(5.6)	

NEWS - National Early Warning Score; CD - clinical deterioration; ARF - acute respiratory failure; CPA - cardiopulmonary arrest; SD - standard deviation; Min - minimum; Max - maximum; TI - tracheal intubation; MV - mechanical ventilation;

* Kruskal-Wallis test;

** chi-square test.

Patients who presented CD at any time during hospitalization had a higher proportion of high risk than those without deterioration, who had a higher percentage of low risk (p=0.0001). Patients with ARF had a higher proportion of high risk when compared to the other risk categories (p<0.0001). In patients who presented more than one CD (p<0.0001), such as ARF (p=0.012), shock (p=0.0001) and CPA (p=0.0105), the proportion of high risk was higher and the proportion of low-medium risk was lower (Table 1).

Patients with low-medium risk were hospitalized for fewer days than those with high risk (p=0.0162). Individuals on mechanical ventilation (p<0.0001), those positioned prone (p=0.0015) and those who died (p=0.0098) had a higher proportion of high risk (Table 1).

Regarding the association of risk categories with NEWS Age, patients categorized in blue/green, and yellow presented a higher proportion of low, low-medium and medium risk than those in the orange and red categories, which presented a higher proportion of high risk (p<0.0001) (Table 2).

**Table 2:** Association of clinical risk, according to the National Early Warning Score Age, with risk category, clinical deterioration, severity markers, prone position and outcomes, São Paulo, São Paulo, Brazil, 2020

	Clinical risk according to NEWS Age n(%)				Total	*p* value
	Low n=29	Low-medium n=14	Medium n=74	High n=239	n=356	
Risk category						
Blue/green	6(12.8)	5(10.6)	15(31.9)	21(44.7)	47(13.2)	<0.0001^***^
Yellow	17(16.5)	2(1.9)	35(34.0)	49(47.6)	103(28.9	
Orange	5(3.7)	7(5.2)	17(12.6)	106(78.5)	135(7.9)	
Red	1(1.4)	-	7(9.9)	63(88.7)	71(19.9)	
CD						
< 24 hours	3(2.4)	4(3.2)	19(15.1)	100(79.4)	126(35.4)	0.0003^**^
> 24 hours	5(6.3)	4(5.1)	13(16.5)	57(72.2)	79(22.2)	
There was no	21(13.9)	6(4.0)	42(27.8)	82(54.3)	151(42.4)	
1st CD - ARF						
Yes	7(3.6)	8(4.1)	31(15.9)	149(76.4)	195(54.8)	0.0001^**^
No	22(13.7)	6(3.7)	43(26.7)	90(55.9)	161(45.2)	
Other CDs						
Yes	6(4.3)	5(3.6)	12(8.7)	115(83.3)	138(38.8)	<0.0001^**^
No	23(10.6)	9(4.1)	62(28.4)	124(56.9)	218(61.2)	
Other CD (ARF)						
Yes	4(4.2)	5(5.3)	9(9.5)	77(81.1)	95(26.7)	0.0020^**^
No	25(9.6)	9(3.4)	65(24.9)	162(62.1)	261(73.3)	
Other CD (shock)						
Yes	5(4.3)	4(3.4)	10(8.6)	97(83.6)	116(32.6)	0.0001^**^
No	24(10.0)	10(4.2)	64(26.7)	142(59.2)	24(67.4)	
Other CD (CPA)						
Yes	4(4.2)	2(2.1)	10(10.4)	80(83.3)	96(27)	0.0013^**^
No	25(9.6)	12(4.6)	64(24.6)	159(61.2)	260(73)	
TI/MV						
Yes	6(4.7)	4(3.1)	12(9.4)	106(82.8)	128(36)	<0.0001^**^
No	23(10.1)	10(4.4)	62(27.2)	133(58.3)	228(64)	
Prone position						
Yes	4(4.5)	3(3.4)	11(12.4)	71(79.8)	89(25)	0.0304^**^
No	25(9.4)	11(4.1)	63(23.6)	168(62.9)	267(75)	
Length of hospitalization						
Mean (SD)	9.59(9.34)	12.2(10.2)	11.2(12.1)	14.67(15.0)	13.46(13.9)	0.0325^*^
Median (min-max)	7(1-40)	9(1-35)	8(0-55)	10(1-98)	8(0-98)	
Final outcome						
Discharge	24(82.8)	11(78.6)	55(74.3)	146(61.1)	236(66.3)	0.0001^***^
Death	5(17.2)	3(21.4)	9(12.2)	83(34.7)	100(28.1)	
Transfer	-	-	10(13.5)	10(4.2)	20(5.6)	

NEWS - National Early Warning Score; CD - clinical deterioration; ARF - acute respiratory failure; CPA - cardiopulmonary arrest; SD - standard deviation; Min - minimum; Max - maximum; TI - tracheal intubation; MV - mechanical ventilation;

* Kruskal-Wallis test;

** chi-square test;

*** likelihood ratio test.

Participants categorized as high clinical risk had a higher proportion of CD in the first 24 hours (p=0.003) and throughout hospitalization (p<0.0001), with the first most frequent CD in the first 24 hours being ARF (p<0.0001), and, throughout hospitalization, were ARF (p=0.0020), shock (p=0.0001) and CPA (p=0.0013) (Table 2).

High-risk patients had a higher proportion of need for mechanical ventilation (p<0.0001), prone positioning (p=0.0304), longer hospital stay (p=0.0325) and death (p=0.0001) (Table 2).

## DISCUSSION

Early Warning Scores, in general, are important components of several Rapid Response Systems spread throughout the world, with the aim of recognizing early signs of CD and triggering the appropriate response, increasing the safety of patients hospitalized in settings where monitoring is not continuous^(15,16)^. However, its performance has been proven in various clinical settings and situations, which was no different during the pandemic^(16,17)^.

Most patients admitted to the ED already had a certain degree of deterioration, since the mean NEWS2 and NEWS Age scores were 6.7 and 7.81, respectively, and were characterized as high risk. In a cohort study conducted in hospitals in the USA, 2,741 patients admitted with a diagnosis of COVID-19 were followed, concluding that ARF was the most prevalent organ dysfunction and that hypoxemia was a predictor of critical illness and mortality^(18)^. Although NEWS2 treated hypoxemia as a binary variable, the Royal College of Physicians recommended that increases in supplemental oxygen supply could trigger medical reassessment and increased surveillance^(19)^.

A retrospective analysis published in the United Kingdom assessing the performance of NEWS2 in COVID-19 admissions showed that NEWS2 with a cut-off point ≥ 5 (intermediate and high risk) was associated with the occurrence of CD^(19)^. Another publication, which compared deterioration scores, found that NEWS2 performed better in predicting the severe form of COVID-19 than other scores applied at the bedside^(20)^. A retrospective cohort study conducted in a tertiary hospital in China with 62,403 patients treated in the ED demonstrated that NEWS2 was significantly better when compared to other early warning scores in predicting admission and death in the ED, in addition to the occurrence of ARF^(21)^.

Regarding NEWS Age, an adaptation implemented in a large health service in China, at the beginning of the pandemic, which adds 3 points for individuals ≥ 65 years old, showed better performance when compared to NEWS2^(22)^. Although the present study did not aim to test the sensitivity and specificity of NEWS2 and NEWS Age, the clinical risk of both scales was associated with unfavorable outcomes, such as ARF, length of hospitalization, days of mechanical ventilation and mortality.

Patients with COVID-19 can rapidly progress to severe respiratory failure, shock and CPA; therefore, early identification and timely treatment of cases are crucial for a good outcome^(23)^. Thus, the use of CD scores, such as NEWS2, at patient admission, demonstrates a high predictive effect for death in the ED, hospitalization and death in the Intensive Care Unit^(24,25)^.

In this study, it was shown that participants categorized in red had higher NEWS2 and NEWS Age than those stratified in the blue/green, yellow and orange categories, as well as mortality, which was higher in individuals in the red group, demonstrating that the protocol correctly categorized patients with possible CD. A prospective cohort study involving 1,010 individuals admitted to an ED in Mexico associated the red and orange categories of the Manchester Triage System with a higher mortality rate^(25)^.

### Study limitations

The study had limitations such as being conducted in a single center and incomplete records of the parameters for calculating alert scores, which limited the number of participants. However, it was conducted in a referral hospital in the capital of São Paulo, for the care of suspected cases of COVID-19 during the pandemic.

Contributions to health, nursing, or public policy

Risk stratification in EDs is essential to identify the urgency of patient care, as well as to recognize the risks of CD in hospitalized patients, and is a challenge to be overcome in the effort to increase patient safety, especially in hospitalized patients in wards. In this scenario, due to overload and high demand, it seems rational to use early warning scores to support and standardize nurses’ clinical assessment and increase health safety.

## CONCLUSIONS

This study concluded that, when assessed by NEWS2 and NEWS Age, high-risk patients were stratified in the red and orange categories, had a higher proportion of CD, the most frequent being ARF, had a greater need for prone positioning and mechanical ventilation, and had a higher proportion of death.

NEWS2 and NEWS Age can be useful in supporting nurses in patient assessment, quickly and objectively identifying possible CDs and enabling the early implementation of measures to prevent harm to patients.

## Data Availability

Not applicable.
