# Supplementary material for: COVID-19: association of the National Early Warning Score with triage categories, severity and outcomes
Source: Rev Bras Enferm. 2025 Aug 8;78(3):e20240069. doi: 10.1590/0034-7167-2024-0069 (PMC12334200; doi:10.1590/0034-7167-2024-0069)
Supplement: Translation

# Translation: COVID-19: associação do *National Early Warning Score* com categorias de triagem, gravidade e desfechos

**DOI:** 10.1590/0034-7167-2024-0069pt

**Authors:** Luiz Humberto Vieri Piacezzi, Jaine Novaes da Silva, Rui Carlos Negrão Baptista, Karina Aparecida Lopes da Costa, Maria Carolina Barbosa Teixeira Lopes, Ruth Ester Assayag Batista, Cássia Regina Vancini Campanharo

**Affiliations:** I Universidade Federal de São Paulo. São Paulo, São Paulo, Brasil; II Escola Superior de Enfermagem de Coimbra. Coimbra, Portugal

**Keywords:** Triagem, Escore de Alerta Precoce, COVID-19, Serviços Médicos de Emergência, Cuidados de Enfermagem.

## Abstract

**Objetivos:**

identificar a associação do *National Early Warning Score* (NEWS2) e NEWS Age com as categorias de risco, marcadores de gravidade e desfechos no serviço de emergência.

**Métodos:**

coorte retrospectiva, realizada em hospital de alta complexidade, com 356 pacientes internados (média de idade 59,4; ±14,4 anos) com COVID-19, de abril a agosto de 2020. Para verificar associação entre as categorias de risco e os escores de alerta, utilizaram-se os testes qui-quadrado, Kruskal-Wallis e razão de verossimilhança (p<0,05).

**Resultados:**

pacientes classificados na categoria vermelha apresentaram NEWS2 e NEWS Age (<0,0001) mais elevados que os demais. O risco clínico categorizado pelos escores foi associado à deterioração clínica (p<0,0001), intubação orotraqueal (p<0,0001) e óbito (NEWS - p=0,0098/NEWS Age - p<0,0001).

**Conclusões:**

pontuações ≥ 7 foram associados à classificação de risco vermelho/laranja, deterioração clínica e ocorrência de óbito.

## INTRODUÇÃO

A pandemia por *Coronavirus Disease* (COVID-19) trouxe grandes desafios para os profissionais de saúde, principalmente para os serviços de emergência (SE), que são porta de entrada para o Sistema Único de Saúde (SUS)^(1)^. Essas unidades enfrentaram superlotação e rápida deterioração clínica dos pacientes^(2)^. Os efeitos negativos da superlotação na qualidade e segurança dos pacientes já são bem estabelecidos e documentados. Coorte retrospectiva, com 995.379 admissões no SE, em 187 hospitais, evidenciou que pacientes atendidos em períodos de superlotação tiveram cinco vezes mais chances de óbito^(3)^.

A infecção por COVID-19 pode variar de simples resfriado até pneumonia grave, levando à síndrome respiratória aguda grave, choque circulatório e morte^(4,5)^. Diante dessa situação, torna-se primordial que a equipe de saúde, especialmente a enfermagem, desenvolva habilidades para realizar avaliação que identifique precocemente a deterioração clínica nesses pacientes, para que medidas terapêuticas sejam adotadas precocemente, prevenindo desfechos desfavoráveis. A utilização dos escores de alerta precoce para deterioração clínica tem demonstrado bom desempenho no ambiente hospitalar, diminuindo a mortalidade e melhorando a sobrevida desses indivíduos^(6,7)^.

O *National Early Warning Score* (NEWS2) e o NEWS Age podem auxiliar na identificação de deterioração clínica em pacientes adultos. Estudos demonstram que a utilização do NEWS2 na admissão do paciente com COVID-19 no SE pode identificar, precocemente, possíveis complicações clínicas associadas à doença^(7,8)^. Entretanto, especialmente no cenário nacional, a literatura é escassa.

Diante do exposto, intensificou-se a preocupação com a qualidade assistencial nos SE, e tornou-se pertinente investigar qual é a relação entre os escores de alerta precoce, as categorias de risco e desfechos dos pacientes com COVID-19, tendo em vista que essas ferramentas minimizam desfechos desfavoráveis e favorecem a segurança em saúde.

## OBJETIVOS

Identificar a relação do NEWS2 e NEWS Age com as categorias de risco, marcadores de gravidade e desfechos no SE.

## MÉTODOS

### Aspectos éticos

O estudo foi norteado pela Resolução n° 466/2012, sendo aprovado pelo Comitê de Ética em Pesquisa da Universidade Federal de São Paulo. O Termo de Consentimento Livre e Esclarecido foi obtido em formato *online*. Os dados de identificação foram anonimizados, sendo substituídos por *tags*.

### Desenho, período e local do estudo

Estudo coorte, retrospectivo, realizado no Hospital São Paulo (HSP), de grande porte e alta complexidade, que atende prioritariamente pacientes do SUS. O HSP é responsável pela cobertura de uma área que abrange mais de cinco milhões de habitantes, além de atender pacientes oriundos de outros estados, e foi referência para o atendimento de casos de COVID-19^(9)^. Os passos metodológicos foram em concordância com as diretrizes do *STrengthening the Reporting of OBservational studies in Epidemiology* (STROBE)^(10)^.

### População: critérios de inclusão e exclusão

A população foi composta por 356 pacientes, maiores de 18 anos, atendidos no SE do HSP, com diagnóstico de COVID-19 e que permaneceram internados de abril a agosto de 2020. Foram excluídos gestantes, pacientes em cuidados paliativos e pacientes com dados incompletos para o cálculo dos escores.

### Protocolo do estudo

Os dados foram obtidos por meio da análise dos prontuários eletrônicos; para tanto, foi elaborado um instrumento de coleta pelo aplicativo *Research Electronic Data Capture* (REDCap^®^), composto por variáveis sociodemográficas, comorbidades, sinais e sintomas, categoria de risco, sinais vitais para cálculo do NEWS2 e NEWS Age na admissão, ocorrência de deterioração clínica (DC) (insuficiência respiratória aguda (IRpA), choque e parada cardiorrespiratória (PCR), marcadores de gravidade (necessidade de prona, ventilação mecânica e tempo de internação) e desfechos), alta, óbito, ou transferência^(11)^.

O Acolhimento com Avaliação e Classificação de Risco no HSP utiliza cinco categorias, que preconizam o tempo de espera recomendado para atendimento médico: vermelho (atendimento imediato); laranja (dez minutos); amarelo (60 minutos); verde (120 minutos); e azul (240 minutos)^(12)^. O NEWS2 é composto por frequência cardíaca e respiratória, saturação de oxigênio, necessidade de suporte de oxigênio, temperatura, pressão arterial sistólica, e nível de consciência^(13)^. No caso do NEWS Age, o cálculo é feito somando-se 3 pontos para pacientes com idade acima de 64 anos^(14)^. Os indivíduos são categorizados em risco clínico baixo (0-4 pontos), baixo-médio (3 em qualquer parâmetro), intermediário (5-6) e alto (≥ 7)^(13)^.

### Análise dos resultados e estatística

Para as variáveis contínuas, calcularam-se média, desvio padrão, mediana, mínimo e máximo. Em relação às variáveis categóricas, calcularam-se frequência e percentual. Para comparar as variáveis categóricas com risco clínico do NEWS2 e NEWS2, foi utilizado o teste qui-quadrado e, quando necessário, o teste da razão de verossimilhança. Para comparar as variáveis contínuas com risco clínico do NEWS2 e NEWS2, foi utilizado o teste de Kruskal-Wallis. Foi utilizado um nível de significância de 5% (valor de p< 0,05). As análises foram realizadas pelo *software Statistical Package for the Social Sciences* ^®^.

## RESULTADOS

A média de idade da população (n=356) foi de 59,4 anos (±14,47), sendo a maioria homem (n=226-63,7%), branca (n=181-51,4%), com ensino fundamental incompleto (n=105-37,4%) e com comorbidades (n=304-85,4%). A comorbidade mais frequente foi hipertensão arterial sistêmica (n=211-59,3%), seguida de Diabetes Mellitus (n=137-38,5%), cardiopatias (n=76-21,3%) e transplante de órgãos sólidos (n=70-19,7%).

Os sintomas mais frequentes foram dispneia (n=246-69,1%), tosse (n=244-68,5%) e febre (n=223-62,6%), sendo que a média de dias de início dos sintomas até a procura pelo atendimento foi de 7,48 (±4,05) dias. Em relação à categoria de risco, 135 (37,9%) pacientes foram classificados na categoria laranja, 103 (28,9%), na amarela, 71 (19,9%), na vermelha, 46 (12,9%), na verde, e um (0,3%), na azul. Quando se calculou o risco clínico na admissão no SE, a maioria apresentou “alto risco” segundo o NEWS2 (n=53,4%) e NEWS Age (n**=**67,1%).

A maior parte dos pacientes (n=205-57,6%) apresentou DC, sendo que, em 61,4% (n=126), esta ocorreu nas primeiras 24 horas após a admissão no SE. A primeira DC mais frequente foi IRpA (n=195-54,8%), e, durante a internação, foi choque (n=116-32,6%). A maioria dos pacientes não necessitou de posição de prona (n=267-75,0%) e ventilação mecânica (n=228-64,0%). A média de dias de internação foi de 13,46 (±13,98) dias. A alta hospitalar (n=236-66,3%) foi o desfecho mais frequente.

Pacientes classificados nas categorias azul/verde e amarela apresentaram maior proporção de baixo e baixo-médio risco pelo NEWS2 do que os classificados nas categorias laranja e vermelha, os quais apresentaram maior proporção de alto risco (p<0,0001) (Tabela 1).

**Tabela 1:** Associação do risco clínico, segundo o *National Early Warning Score*, à categoria de risco, deterioração clínica, marcadores de gravidade e aos desfechos, São Paulo, São Paulo, Brasil, 2020

	Risco clínico segundo NEWS2 n(%)				Total	Valor de *p*
	Baixo n=45	Baixo-médio n=23	Médio n=98	Alto n=190	n=356	
Categoria de risco						
Azul/verde	8(17,8)	9(19,1)	18(38,3)	12(25,5)	47(13,2)	<0,0001^**^
Amarela	27(26,2)	5(4,9)	40(38,8)	31(30,1)	103(28,9)	
Laranja	8(5,9)	9(6,7)	28(20,7)	90(66,7)	135(7,9)	
Vermelha	2(2,8)	-	12(16,9)	57(80,3)	71(19,9)	
DC						
Sim < 24 horas	6(4,8)	6 (4,8)	28(22,2)	86(68,3)	126(35,4)	0,0001^**^
Sim > 24 horas	7(8,9)	6(7,6)	23(29,1)	43(54,4)	79(22,2)	
Não	32(21,2)	11(7,3)	47(31,1)	61(40,4)	151(42,4)	
1ª DC - IRpA						
Sim	11(5,6)	12(6,2)	51(26,2)	121(62,1)	195(54,8)	<0,0001^**^
Não	34(21,1)	11(6,8)	47(29,2)	69(42,9)	161(45,2)	
Outras DC						
Sim	10(7,2)	7(5,1)	23(16,7)	98(71,0)	138(38,8)	<0,0001^**^
Não	35(16,1)	16(7,3)	75(34,4)	92(42,2)	218(61,2)	
Outras DC (IRpA)						
Sim	8(8,4)	6(6,3)	17(17,9)	64(67,4)	95(26,7)	0,012^**^
Não	37(14,2)	17(6,5)	81(31,0)	126(48,3)	261(73,3)	
Outras DC (choque)						
Sim	8(6,9)	6(5,2)	20(17,2)	82(70,7)	116(32,6)	0,0001^*^
Não	37(15,4)	17(7,1)	78(32,5)	108(45,0)	24(67,4)	
Outras DC (PCR)						
Sim	7 (7,3)	4 (4,2)	20 (20,8)	65 (67,7)	96(27)	0,0105^**^
Não	38 (14,6)	19 (7,3)	78 (30,0)	125 (48,1)	260(73)	
Dias de internação						
Média (DP)	9,6 (8,5)	12,17 (9,9)	11,5 (13,1)	15,5 (15,5)	13,4 (13,9)	0,0162^*^
Mediana (mín-máx)	7(1-40)	9(1-37)	8(0-80)	11(1-98)	8(0-98)	
IOT/VM						
Sim	10 (7,8)	6 (4,7)	21 (16,4)	91 (71,1)	128(36)	<0,0001^**^
Não	35 (15,4)	17 (7,5)	77 (33,8)	99 (43,4)	228(64)	
Posição prona						
Sim	5 (5,6)	5 (5,6)	16 (18,0)	63 (70,8)	89(25)	0,0015^**^
Não	40 (15,0)	18 (6,7)	82 (30,7)	127 (47,6)	267(75)	
Desfecho final						
Alta	36 (15,3)	18 (7,6)	70 (29,7)	112 (47,5)	236(66,3)	0,0098^**^
Óbito	8 (8,0)	5 (5,0)	19 (19,0)	68 (68,0)	100(28,1)	
Transferência	1 (5,0)	-	9 (45,0)	10 (50,0)	20(5,6)	

NEWS - National Early Warning Score; DC - deterioração clínica; IRpA - insuficiência respiratória aguda; PCR - parada cardiorrespiratória; DP - desvio padrão; Mín - mínimo; Máx - máximo; IT - intubação traqueal; VM - ventilação mecânica;

* teste de Kruskal-Wallis;

** teste qui-quadrado.

Pacientes que apresentaram DC, em quer momento da internação, apresentaram maior proporção de alto risco do que aqueles sem deterioração, que apresentaram maior percentual de baixo risco (p=0,0001). Pacientes com IRpA apresentaram maior proporção de alto risco quando comparados às demais categorias de risco (p<0,0001). Em pacientes que apresentaram mais de uma DC (p<0,0001), como IRpA (p=0,012), choque (p=0,0001) e PCR (p=0,0105), a proporção de alto risco foi maior e menor de baixo-médio risco (Tabela 1).

Os pacientes com baixo-médio risco ficaram internados por menos dias que aqueles com alto risco (p=0,0162). Os indivíduos em ventilação mecânica (p<0,0001), aqueles posicionados em prona (p=0,0015) e os que evoluíram para óbito (p=0,0098) apresentaram maior proporção de alto risco (Tabela 1).

Em relação à associação das categorias de risco com NEWS Age, pacientes categorizados nas cores azul/verde e amarela apresentaram maior proporção de baixo, baixo-médio e médio risco do que os das categorias laranja e vermelha, que apresentaram maior proporção de alto risco (p<0,0001) (Tabela 2).

**Tabela 2:** Associação do risco clínico, segundo o *National Early Warning Score* Age, à categoria de risco, deterioração clínica, marcadores de gravidade, posição prona e desfechos, São Paulo, São Paulo, Brasil, 2020

	Risco clínico segundo NEWS Age n(%)				Total	Valor de *p*
	Baixo n=29	Baixo-médio n=14	Médio n=74	Alto n=239	n=356	
Categoria de risco						
Azul/verde	6(12,8)	5(10,6)	15(31,9)	21(44,7)	47(13,2)	<0,0001^***^
Amarelo	17(16,5)	2(1,9)	35(34,0)	49(47,6)	103(28,9	
Laranja	5(3,7)	7(5,2)	17(12,6)	106(78,5)	135(7,9)	
Vermelho	1(1,4)	-	7(9,9)	63(88,7)	71(19,9)	
DC						
< 24 horas	3(2,4)	4(3,2)	19(15,1)	100(79,4)	126(35,4)	0,0003^**^
> 24 horas	5(6,3)	4(5,1)	13(16,5)	57(72,2)	79(22,2)	
Não houve	21(13,9)	6(4,0)	42(27,8)	82(54,3)	151(42,4)	
1ª DC - IRpA						
Sim	7(3,6)	8(4,1)	31(15,9)	149(76,4)	195(54,8)	0,0001^**^
Não	22(13,7)	6(3,7)	43(26,7)	90(55,9)	161(45,2)	
Outras DC						
Sim	6(4,3)	5(3,6)	12(8,7)	115(83,3)	138(38,8)	<0,0001^**^
Não	23(10,6)	9(4,1)	62(28,4)	124(56,9)	218(61,2)	
Outras DC (IRpA)						
Sim	4(4,2)	5(5,3)	9(9,5)	77(81,1)	95(26,7)	0,0020^**^
Não	25(9,6)	9(3,4)	65(24,9)	162(62,1)	261(73,3)	
Outras DC (choque)						
Sim	5(4,3)	4(3,4)	10(8,6)	97(83,6)	116(32,6)	0,0001^**^
Não	24(10,0)	10(4,2)	64(26,7)	142(59,2)	24(67,4)	
Outras DC (PCR)						
Sim	4(4,2)	2(2,1)	10(10,4)	80(83,3)	96(27)	0,0013^**^
Não	25(9,6)	12(4,6)	64(24,6)	159(61,2)	260(73)	
IOT/VM						
Sim	6(4,7)	4(3,1)	12(9,4)	106(82,8)	128(36)	<0,0001^**^
Não	23(10,1)	10(4,4)	62(27,2)	133(58,3)	228(64)	
Posição prona						
Sim	4(4,5)	3(3,4)	11(12,4)	71(79,8)	89(25)	0,0304^**^
Não	25(9,4)	11(4,1)	63(23,6)	168(62,9)	267(75)	
Dias de internação						
Média (DP)	9,59(9,34)	12,2(10,2)	11,2(12,1)	14,67(15,0)	13,46(13,9)	0,0325^*^
Mediana (mín-máx)	7(1-40)	9(1-35)	8(0-55)	10(1-98)	8(0-98)	
Desfecho final						
Alta	24(82,8)	11(78,6)	55(74,3)	146(61,1)	236(66,3)	0,0001^***^
Óbito	5(17,2)	3(21,4)	9(12,2)	83(34,7)	100(28,1)	
Transferência	-	-	10(13,5)	10(4,2)	20(5,6)	

NEWS - National Early Warning Score; DC - deterioração clínica; IRpA - insuficiência respiratória aguda; PCR - parada cardiorrespiratória; DP - desvio padrão; Mín - mínimo; Máx - máximo; IT - intubação traqueal; VM - ventilação mecânica;

* teste de Kruskal-Wallis;

** teste qui-quadrado;

*** teste da razão de verossimilhança.

Participantes categorizados como risco clínico alto apresentaram maior proporção de DC nas primeiras 24 horas (p=0,003) e ao longo da internação (p<0,0001), sendo que, nas primeiras 24 horas, a primeira DC mais frequente foi IRpA (p<0,0001), e, ao longo da internação, foram IRpA (p=0,0020), choque (p=0,0001) e PCR (p=0,0013) (Tabela 2).

Pacientes com alto risco tiveram maior proporção de necessidade de ventilação mecânica (p<0,0001), posicionamento em prona (p=0,0304), maior tempo de internação (p=0,0325) e óbito (p=0,0001) (Tabela 2).

## DISCUSSÃO

Os *Early Warning Scores*, em geral, são componentes importantes de diversos Sistemas de Resposta Rápida difundidos pelo mundo, com o objetivo de reconhecer precocemente sinais de DC e acionar a resposta adequada, aumentando a segurança de pacientes internados em ambientes onde a monitorização não é contínua^(15,16)^. No entanto, seu desempenho foi comprovado em diversos cenários e situações clínicas, o que não foi diferente durante a pandemia^(16,17)^.

A maioria dos pacientes admitidos no SE já apresentava um determinado grau de deterioração, uma vez que a pontuação média do NEWS2 e do NEWS Age foi de 6,7 e 7,81, respectivamente, sendo caracterizados como alto risco. Em coorte realizada em hospitais nos EUA, 2.741 pacientes internados com diagnóstico de COVID-19 foram acompanhados, concluindo-se que a IRpA era disfunção orgânica mais prevalente e que hipoxemia era preditor da doença crítica e mortalidade^(18)^. Embora o NEWS2 tratasse a hipoxemia como variável binária, a *Royal College of Physicians* recomendou que acréscimos no fornecimento de oxigênio suplementar pudessem desencadear reavaliação médica e aumento na vigilância^(19)^.

Análise retrospectiva, publicada no Reino Unido, avaliando o desempenho do NEWS2 nas admissões por COVID-19, mostrou que o NEWS2 com ponto de corte ≥ 5 (intermediário e alto risco) foi associado à ocorrência de DC^(19)^. Outra publicação, que comparou escores de deterioração, identificou que o NEWS2 teve melhor desempenho para prever a forma grave da COVID-19 em relação aos demais escores aplicados à beira-leito^(20)^. Estudo de coorte retrospectivo, realizado em hospital terciário na China com 62.403 pacientes atendidos no SE, demonstrou que o NEWS2 foi significativamente melhor quando comparado a outros escores de alerta precoce na previsão de admissão e óbito no SE, além de ocorrência de IRpA^(21)^.

Em relação ao NEWS Age, uma adaptação implementada em um grande serviço de saúde na China, no início da pandemia, que acrescenta 3 pontos para indivíduos ≥ 65 anos, mostrou melhor desempenho quando comparado ao NEWS2^(22)^. Apesar de o presente estudo não ter o objetivo de testar a sensibilidade e a especificidade do NEWS2 e do NEWS Age, o risco clínico de ambas as escalas foi associado para desfechos desfavoráveis, como o DC IRpA, dias de internação, dias de ventilação mecânica e mortalidade.

Pacientes com COVID-19 podem evoluir rapidamente para insuficiência respiratória grave, choque e PCR; logo, a identificação precoce e o tratamento oportuno dos casos são cruciais para um bom desfecho^(23)^. Destarte, a utilização de escores de DC, como o NEWS2, na admissão do paciente, demonstra um alto efeito preditivo para óbito no SE, internação e óbito na Unidade de Terapia Intensiva^(24,25)^.

Neste estudo, foi evidenciado que os participantes categorizados em vermelho apresentaram NEWS2 e NEWS Age mais elevados do que aqueles classificados nas categorias azul/verde, amarelo e laranja, assim como a mortalidade, que foi maior nos indivíduos do grupo vermelho, demonstrando que o protocolo categorizou corretamente os pacientes com possível DC. Estudo de coorte prospectivo, envolvendo 1.010 indivíduos admitidos em SE no México, associou as categorias vermelho e laranja do Sistema de Triagem Manchester a uma maior taxa de mortalidade^(25)^.

### Limitações do estudo

O estudo teve como limitações a realização em centro único e a incompletude dos registros dos parâmetros para cálculo dos escores de alerta, o que limitou o número de participantes. Entretanto, foi realizado em hospital de referência, na capital paulista, para atendimento dos casos suspeitos de COVID-19 durante a pandemia.

### Contribuições para as áreas da saúde, enfermagem, ou políticas públicas

A classificação de risco nos SE é fundamental para identificar a urgência do atendimento dos pacientes, bem como reconhecer os riscos de DC em pacientes internado, sendo um desafio a ser vencido no esforço de aumentar a segurança do paciente, especialmente internado em enfermarias. Nesse cenário, devido à sobrecarga e alta demanda, parece racional a utilização de escores de alerta precoce para subsidiar e padronizar a avaliação clínica do enfermeiro e aumentar a segurança em saúde.

## CONCLUSÕES

Este estudo concluiu que, quando avaliados pelo NEWS2 e NEWS Age, pacientes com alto risco foram classificados nas categorias vermelha e laranja, apresentaram maior proporção de DC, sendo a mais frequente a IRpA, tiveram mais necessidade de posicionamento em prona e ventilação mecânica, e tiveram maior proporção de óbito.

O NEWS2 e o NEWS Age podem ser úteis para subsidiar os enfermeiros na avaliação do paciente, identificando, de maneira rápida e objetiva, possíveis DC e possibilitando a implementação precoce de medidas para evitar agravos ao paciente.

## Data Availability

Não se aplica.
